# Supplementary material for: WordCommentsAnalyzer: A windows software tool for qualitative research
Source: F1000Res. 2018 Sep 4;7:536. Originally published 2018 May 3. [Version 2] doi: 10.12688/f1000research.14819.2 (PMC6137411; doi:10.12688/f1000research.14819.2)
Supplement: Supplementary file 3 [file f1000research-7-17614-s0002.tgz › b2a79496-4ada-47c4-9682-a99ce867836d.zip!/Eight Iranian Health Journals/Journal of Education and Community Health_8.docx]

© Journal of Education and Community Health. This work is licensed under CC BY-NC 4.0 (https://creativecommons.org/licenses/by-nc/4.0/).

Volume & Issue: دوره 3 - شماره 1

Title: اثربخشی برنامه آموزشی مبتنی بر وب در پیشگیری از مصرف سیگار در نوجوانان پسر: کاربرد مدل تصورات و تمایلات

http://jech.umsha.ac.ir/article-1-194-fa.html

سابقه و هدف: بکارگیری استراتژی‌های مؤثر آموزشی در پیشگیری از مصرف سیگار در کشورهای در حال توسعه کشورها ضروری به نظر می‌رسد. بنابراین، مطالعه حاضر باهدف تعیین تأثیر مداخله آموزشی مبتنی بر وب در پیشگیری از مصرف سیگار در نوجوانان پسر شهر همدان با بهره‌گیری از مدل تصورات و تمایلات انجام گردید. مواد و روش ها: در این مطالعه مداخله ای، 114 نفر از نوجوانان پسر شهر همدان به‌صورت تصادفی به دو گروه آزمون و کنترل تقسیم ‌شده بودند. ابزار گردآوری اطلاعات شامل پرسشنامه مبتنی بر مدل تصورت و تمایلات و اطلاعات جمعیت‌شناختی آزمودنی‌ها بود. برنامه مداخله‌ای شامل 11 جلسه آموزشی مبتنی بر وب بود که در مدت زمان 2 ماه برای شرکت‌کنندگان گروه مداخله به مرحله اجرا درآمد. دو گروه آزمون و کنترل شش ماه پس از مداخله مورد پیگیری قرار گرفتند. داده‌ها با استفاده از نرم‌افزارهای SPSS-18 و با بکارگیری آزمون کای‌اسکوئر، تی‌مستقل، تی‌زوجی و کاپا مورد تجزیه ‌و تحلیل قرار گرفتند.    یافته ها: اختلاف های معنی‌داری برای میانگین نمرات نگرش، هنجارهای انتزاعی، تصورات، قصد و اشتیاق رفتاری بین گروه‌های آزمون و کنترل بعد از اجرای برنامه مداخله‌ای مشاهده گردید (0.05P<). مصرف سیگار در گروه آزمون کاهش یافته بود، ولیکن این کاهش از نظر آماری معنی‌دار نبود (0.392P=). نتیجه‌گیری: نتایج مطالعه حاضر نشان داد که بکارگیری مداخلات مبتنی بر وب، استراتژی آموزشی مؤثری در پیشگیری از افزایش احتمالی مصرف سیگار در بین نوجوانان پسر می‌باشد.

Title: تاثیر مداخله آموزشی اصلاح سبک زندگی بر کنترل فشار خون در بیماران مبتلا به پرفشاری خون

http://jech.umsha.ac.ir/article-1-188-fa.html

سابقه و هدف: امروزه بیماری های مزمن از جمله فشارخون بالا رو به افزایش است و این بیماری با سبک زندگی افراد رابطه تنگاتنگی دارد. لذا مطالعه حاضر با هدف سنجش تأثیر مداخله آموزشی سبک زندگی بر کنترل فشارخون در بیماران مبتلا به پرفشاری خون انجام شد. مواد و روش ها: پژوهش حاضر یک مطالعه نیمه تجربی بود که در سال 1394 بر روی 210 نفر از بیماران مبتلا به پرفشاری خون در خانه های بهداشت شهرستان شبستر اجرا گردید. بیماران به صورت تصادفی چندمرحله ای در دو گروه آزمون (105=n) و کنترل (105=n) قرار گرفتند. داده ها با استفاده از پرسشنامه سبک زندگی استاندارد (LSQ) در دو مرحله قبل و 2 ماه پس از مداخله آموزشی گردآوری گردید. جهت تجزیه و تحلیل داده ها از شاخص های آمار توصیفی و آزمون های کای اسکوئر، تی زوجی و تی مستقل استفاده شد. یافته ها: پس از مداخله، میانگین نمرات متغیرهای ورزش، کنترل وزن و تغذیه و سلامت روانی در گروه مداخله به طوری معنی داری افزایش یافته بود (0.05>P)، اما تغییرات انجام شده در گروه کنترل معنی دار نبود (0.05<P). همچنین مداخله آموزشی تاثیر معنی داری در کاهش فشارخون افراد گروه آزمون در مقایسه با گروه کنترل داشته است (0.05>P). نتیجه گیری: طبق یافته ها، مداخله آموزشی در ارتقاء فعالیت فیزیکی، بهبود کنترل وزن و تغذیه و بهبود سلامت روانی در بیمان مبتلا به پرفشاری خون موثر بوده است. همچنین نتایج نشان داد که با بهبود سبک زندگی می توان فشارخون را نیز کنترل کرد.

Title: بررسی مراقبت های دهان و دندان و موانع مربوط به آن در زنان باردار شهر همدان

http://jech.umsha.ac.ir/article-1-168-fa.html

سابقه و هدف: رفتارهای بهداشت دهان و دندان در دوران بارداری به دلیل پیامدهای آن بر سلامت مادر و کودک اهمیت دارد. این پژوهش با هدف بررسی نحوه انجام این رفتارها در زنان باردار و موانع تاثیر گذار بر آنها انجام گرفت. مواد و روش: این مطالعه تحلیلی- مقطعی با مشارکت 280 نفر از زنان باردار شهر همدان در سال 1391 انجام شد. روش نمونه گیری خوشه ای طبقه ای و روش جمع‎آوری داده ها، استفاده از پرسشنامه شامل مشخصات جمعیت شناختی، رفتار مراجعه به دندانپزشک و مسواک زدن و موانع مربوط به انجام این دو رفتار بود. پایایی و روایی ابزار از طریق بررسی همبستگی درونی و مراجعه به پانل متخصصین مورد ارزیابی قرار گرفت. داده ها با استفاده از نرم افزار SPSS نسخه 16 مورد تحلیل قرار گرفتند. یافته ها: میانگین سنی زنان مورد بررسی (4.88±(27 سال بود. میزان مسواک زدن بعد از هر وعده غذایی و استفاده از دهانشویه به ترتیب 12 درصد و 20 درصد بود. نیمی از زنان باردار در طی بارداری اخیر خود به دندانپزشک مراجعه نکرده بودند. خستگی و بی حوصلگی مهم ترین موانع مسواک زدن و هزینه بالا، احساس عدم نیاز و استرس آور بودن محیط دندانپزشکی تأثیرگذارترین موانع عدم مراجعه به دندانپزشکی بودند. عوامل مرتبط در عدم مراجعه به دندانپزشک شامل وضعیت اقتصادی ضعیف (0.011P=) و کار خارج از منزل (0.045P=) بود. نتیجه گیری: با توجه به اهمیت پیامدهای مربوط به مشکلات دهان و دندان در دوران بارداری، توصیه می شود در جهت رفع موانع انجام رفتارهای مراقبتی برنامه ریزی شود.

Title: رابطه اجتناب تجربه ای، جو عاطفی خانواده و خودکنترلی با اعتیاد به بازی های آنلاین در دانشجویان دانشگاه شهید چمران اهواز

http://jech.umsha.ac.ir/article-1-165-fa.html

سابقه و هدف: اعتیاد به بازی­های آنلاین، به عنوان رفتاری وسواسی، افراطی، غیر قابل کنترل و از لحاظ جسمانی و روانی مخرب در نظر گرفته می شود. پژوهش حاضر به منظور تعیین رابطه اجتناب تجربه ای، جو عاطفی خانواده و خودکنترلی با اعتیاد به بازی های آنلاین در دانشجویان دانشگاه شهید چمران اهواز انجام گرفت. مواد و روش ها:  طرح پژوهش حاضر توصیفی و از نوع مطالعات همبستگی می باشد که بر روی 200 نفر  از دانشجویان کارشناسی دانشگاه شهید چمران اهواز که به روش نمونه گیری تصادفی چند مرحله ای انتخاب شدند، انجام شد. اطلاعات جمع آوری شده از طریق نرم افزار SPSS16 و با بکارگیری ضریب همبستگی پیرسون و رگرسیون از نوع همزمان مورد تجزیه و تحلیل قرار گرفت. یافته ها: طبق یافته ها، بین جو عاطفی خانواده و اعتیاد به بازی های آنلاین (r=0.53, P<0.001) و همچنین بین اجتناب تجربه ای و اعتیاد به بازی های آنلاین (r=0.27, P<0.001) همبستگی مثبت و معنادار و بین اعتیاد به بازی های آنلاین و خودکنترلی (r=-0.17, P<0.001) همبستگی منفی و معنادار وجود دارد. همچنین تجزیه و تحلیل رگرسیون نشان داد که جوعاطفی خانواده می­ تواند 28 درصد از واریانس اعتیاد به بازی های آنلاین را تبیین کند. نتیجه گیری: با توجه به نتایج این پژوهش می توان با طراحی مداخلات مناسب از جمله آگاهی دادن به دانشجویان و دست اندرکاران در زمینه علائم و عواقب اعتیاد به بازی های آنلاین و سعی در جهت دهی صحیح استفاده از اینترنت در میان کاربران، به پیشگیری و کنترل موثر این اختلال پرداخت.

Title: تأثیر تمرین ترکیبی بر تراکم استخوان زنان پیش یائسه

http://jech.umsha.ac.ir/article-1-191-fa.html

سابقه و هدف: بهترین راه پیشگیری از بروز پوکی استخوان در سالمندی جلوگیری از کاهش تراکم استخوانی و یا تلاش در جهت حفظ آن در سنین جوانی است، بنابراین هدف از تحقیق حاضر، تاثیر تمرین ترکیبی بر تراکم استخوان زنان پیش­ یائسه بود. مواد و روش‌ها: این تحقیق نیمه ­تجربی، طرح پیش ­آزمون-پس ­آزمون و نوع کاربردی بود. 20 زن پیش­ یائسه 40 تا 45 ساله شهر همدان در سال 94 در دو گروه 10 نفری (گروه تمرین ترکیبی و گروه کنترل) تقسیم شدند. گروه­ تجربی 12 هفته تمرین ترکیبی (شش هفته تمرین در آب و شش هفته تمرین در خشکی)، را سه بار در هفته و هر جلسه 70 دقیقه انجام دادند. تراکم استخوان ران (کل هیپ و سر فمور) آزمودنی ­ها قبل و پس از 12 هفته توسط دستگاه سنجش تراکم استخوان دگزا مورد سنجش قرار گرفت و با استفاده از آمار توصیفی، تی تست مستقل و زوجی و تحلیل کوواریانس در سطح معناداری کمتر از 0.05 با استفاده از نرم افزار SPSS نسخه 21 تحلیل شد. یافته‌ها: تراکم استخوان ران (کل هیپ و گردن فمور) در گروه تمرین ترکیبی در مقایسه با گروه کنترل اختلاف معناداری را نشان داد (0.05>P). پس از 12 هفته در گروه تمرین ترکیبی افزایش تراکم استخوان و در گروه کنترل کاهش تراکم استخوان (0.05>P) مشاهده شد. نتیجه ­گیری: با توجه به این­که تفاوت در بین گروه ­های تمرینی ترکیبی و گروه کنترل مشاهده شد، تمرینات ترکیبی به زنان پیش ­یائسه توصیه می­ شود، زیرا باعث جلوگیری از کاهش تراکم استخوان ناشی از افزایش سن در این دوران می­ شود.

Title: اثربخشی آموزش گروهی مبتنی بر روش باکروید بر بهبود نگرش خوردن و افزایش عزت نفس زنان مبتلا به چاقی رده سنی 30-20 ساله شهر اصفهان

http://jech.umsha.ac.ir/article-1-189-fa.html

سابقه و هدف: رشد روزافزون چاقی در جهان سبب پیدایش مشکلات گوناگون جسمی و روان شناختی برای افراد شده است. لذا پژوهش حاضر با هدف تعیین اثربخشی آموزش گروهی مبتنی بر روش باکروید بر نگرش خوردن و افزایش عزت نفس زنان مبتلا به چاقی رده سنی 30-20 ساله شهر اصفهان انجام شد. مواد و روش ها: این پژوهش به صورت نیمه‌آزمایشی با پیش‌آزمون، پس‌آزمون و پیگیری با گروه گواه صورت گرفت. جامعه پژوهش شامل کلیه زنان مبتلا به چاقی شهر اصفهان در بهار سال 1393 بودند که براساس روش نمونه‌گیری هدفمند 30 نفر از زنان چاق مراجعه‌کننده به کلینیک سلامت سپاهان انتخاب شده و سپس به صورت تصادفی در دو گروه 15 نفری (آزمایش و گواه) جایگزین شدند. مداخلات درمانی مبتنی بر روش باکروید بر روی گروه آزمایش، در طی 16 جلسه دوساعته و هفته‌ای دوبار انجام گرفت. ابزار اندازه‌گیری این پژوهش مقیاس BMI، فرم اطلاعات دموگرافیک، پرسش‌نامه نگرش خوردن و پرسشنامه عزت نفس بودند. در این پژوهش جهت آزمودن فرضیه‌ها از روش تحلیل واریانس با اندازه‌گیری مکرر با بهره گیری از بسته نرم افزاری SPSS-20 استفاده شد. یافته ­ها: نتایج نشان داد که آموزش گروهی مبتنی بر روش باکروید در بهبود عزت نفس تاثیر معنی­دار داشته (0.001P<)، در حالیکه تاثیر معنی­ داری بر بهبود نگرش خوردن در شرکت کنندگان گروه مداخله مشاهده نشد (0.05<P). نتیجه گیری: بنابر یافته­ های حاصل از این پژوهش می­ توان از روش باکروید در جهت افزایش عزت نفس زنان مبتلا به چاقی بهره برد.

Title: اولویت‌های نیاز آموزشی سلامت جوانان بر پایه نیاز سنجی و مشارکت ذی نفعان درحوزه‌های تحت پوشش معاونت بهداشتی دانشگاه علوم پزشکی ایران

http://jech.umsha.ac.ir/article-1-203-fa.html

سابقه و هدف: اولین قدم در برنامه‌ریزی آموزشی شناسایی و اولویت‌بندی نیازهای آموزشی است که نقطه آغاز آن نیازسنجی آموزشی می‌باشد. لذا مطالعه حاضر با هدف نیازسنجی و تعیین اولویت های آموزشی سلامت جوانان تحت پوشش معاونت بهداشت دانشگاه علوم پزشکی ایران انجام گردید. مواد و روش‌ها: پژوهش حاضر یک مطالعه توصیفی و از نوع مقطعی بود که در بین 2842 نفر از جوانان 18 تا 29 ساله تحت پوشش معاونت بهداشت دانشگاه علوم پزشکی ایران در سال 1393 انجام گردید. از روش نمونه گیری دو مرحله‌ای به صورت ابتدا نمونه‌ گیری خوشه‌ای از مراکز تحت پوشش و سپس نمونه گیری تصادفی از جوانان آن مرکز استفاده شده است. ابزار نمونه گیری، پرسشنامه طراحی شده توسط کارشناسان وزارت بهداشت بوده و شاخص‌های آمار توصیفی مورد استفاده قرار گرفت. یافته‌ها: بر اساس امتیازهای حاصل از پرسشنامه، مهمترین نیاز آموزشی سلامت در تمامی مراکز تحت پوشش آموزش مهارت‌های زندگی است (3.34±4.47). مهمترین خدمات بهداشتی اولیه مورد نیاز، معاینات دوره‌ای (0.57±1.38) و بیشترین خدمات مشاوره‌ای مورد نیاز، مهارت‌های قبل از ازدواج (1.89±2.82) است. در اکثر موارد آموزش و اطلاع رسانی ارجح، از طریق خانواده ذکر شده است. نتیجه‌گیری: حل مشکلات جوانان در جامعه، وظیفه هر نظام بهداشتی و درمانی است. برای این کار باید در ابتدا مشکلات شناسایی شده و سپس با توجه به منابع موجود و تعیین اولویتها، برای هر اولویت، برنامه های آموزشی لازم مشخص شود. آنچه در این فرآیند اهمیت دارد، توافق تمامی ذینفعان در مورد لیست اولویت‌ها و برنامه‌ها مدون می‌باشد.

Title: بررسی تاثیر برنامه مداخلات آموزشی در افزایش غربالگری ماموگرافی: یک مرور نظام‌مند

http://jech.umsha.ac.ir/article-1-180-fa.html

سابقه و هدف: سرطان پستان 30 درصد سرطان‌ها را به خود اختصاص داده و دومین عامل مرگ در زنان است. سازمان جهانی بهداشت دو شاخص در پیشگیری از سرطان پستان را پیشنهاد کرده است. یکی آموزش مناسب و دیگری انجام غربالگری که به عنوان سریع‌ترین راه تشخیصی قبل از بروز علائم می‌باشد. لذا این مطالعه مرور نظام‌مند جهت ارزیابی مداخلات آموزشی طراحی شده در زمینه افزایش غربالگری ماموگرافی انجام گردید. مواد و روش‌ها: جستجوی الکترونیکی از پایگاه‌های  Embase, Google Scholar, Scopus, PubMed, Web of Science  و Cochrane و بانک‌های اطلاعات علمی SID، Iran Medex وIrandoc  از ابتدای دی ماه 1388 تا پایان بهمن 1394 و مطالعاتی که علاوه بر داشتن معیارهای ورود بر جزئیات مداخلات آموزشی اشاره شده بود، انتخاب گردید. یافته‌ها: بیشتر مطالعات به صورت کارآزمایی بالینی شاهددار بود. مداخلات بیشتر به شکل آموزش، انگیزش و حمایت و در مدت زمان متفاوت انجام شده بود. نحوه جمع‌آوری داده‌ها به صورت خود گزارش‌دهی و پیامد مورد انتظار انجام غربالگری ماموگرافی بود. نتیجه‏ گیری: شناسایی عوامل مؤثر بر عدم انجام ماموگرافی و استفاده از مداخلات مناسب و استراتژی‌های مؤثر در حوزه‌های آگاهی، نگرش و رفتار منجر به شناسایی و درمان به موقع سرطان پستان خواهد شد.
